# Active screening of presumptive tuberculosis cases in rural communities through youth club members in India: logic model development and feasibility study

**DOI:** 10.3389/fpubh.2025.1713220

**Published:** 2026-01-21

**Authors:** Praveen Kumar Anand, Manjula Singh, Amber Kumar, Seema Sahay, Dhruvendra Pandey, Hitesh Tiwari, Anurag Pappu, V. N. R. Das, Sarika Gupta, Sampada Bangar, Brajraj Ghosh, Ashiq Magrey

**Affiliations:** 1 National Institute for Implementation Research on Non Communicable Diseases, Indian Council of Medical Research, Jodhpur, India; 2 Indian Council of Medical Research, New Delhi, India; 3 All India Institute of Medical Sciences Bhopal, Bhopal, India; 4 ICMR - National Institute of Translational Virology and AIDS Research, Pune, India; 5 Dr. Laxminarayan Pandey Government Medical College Ratlam, Ratlam, India; 6 Model Rural Health Research Unit, Bhanpur Kalan, Jaipur, India; 7 ICMR - Rajendra Memorial Research Institute of Medical Sciences, Patna, India; 8 Department of Pediatrics, King George’s Medical University, Lucknow, India

**Keywords:** active screening, community participation, India, qualitative research, tuberculosis, volunteers

## Abstract

**Introduction:**

The majority of presumptive TB cases report late. Early detection of tuberculosis (TB) cases is crucial for its control. We developed a logic model program involving members of a governmental and community-driven youth club of Nehru Yuva Kendra Sangathan (NYKS) volunteers and explored its feasibility.

**Methodology:**

The study defined the purpose, scope, and program domains. The domains were input, process, output, and outcome. These domains were drafted, refined, and finalized using standard techniques. A qualitative study, including in-depth interviews and focus group discussions, was undertaken with community members, healthcare staff, and NYKS volunteers across diverse settings. Acceptability, feasibility, effectiveness, barriers, and improvement strategies of the developed model were synthesized through thematic analysis.

**Results:**

The developed “Logic Model” aims to actively screen and motivate individuals with TB-suggestive symptoms for early detection of active TB, using various domains and corresponding activities. Information, education, communication (IEC), screening, referral, and case detection activities have been monitored using 36 indicators. Most participants expressed acceptance of the model, owing to its alignment with community values, trust among volunteers, and perceived benefits. Key motivators included the proximity of services, improved awareness, and civic responsibility. Barriers included a lack of financial incentives, logistical challenges, and overlapping stakeholder roles. Suggestions for improvement included enhancing gender diversity, confidentiality, volunteer training, and intersectoral coordination.

**Discussion:**

The developed logic model provides a visual display of the input, process, output, and outcome domains, their activities, and relationships. The model links resources, activities, and outcomes for the screening and referral of diagnosed cases. It was broadly accepted, despite some reported challenges. Addressing barriers is essential for program sustainability. Tailored strategies to improve volunteer support, training, and trust-building can enhance the model’s effectiveness and contribute to India’s TB elimination program.

## 1 Introduction

India is one of the most affected countries by tuberculosis. The WHO Global Tuberculosis Report 2024 found that India alone accounted for 26% of global cases in 2023 (1). The Government of India formulated the National Strategic Plan (NSP 2017–25) for TB elimination by the year 2025 with ambitious and innovative measures (2). However, it was realized by 2020 that the NSP-2017–25 was unlikely to achieve its objectives (3). Consequently, NSP India 2025 was introduced in 2020, which reasserts the focus on early diagnosis along with suitable patient support systems (3). A significant focus of research in the NSP 2017–2025 is dedicated to identifying the most effective strategies for detecting TB cases early (2). NSP 2025 advocates for undertaking research on case finding, among other strategies (3).

Studies have found that communities and patients play a crucial role in the early identification of TB cases (4–7). However, there is a felt need for implementation research model studies to effectively operationalize and integrate community workers into existing health systems, ensuring scalability and sustainability (8, 9).

This study developed a logic model, engaging youth clubs under the Nehru Yuva Kendra Sangathan (NYKS) as a platform for community participation in TB case finding and explored deeper insights into the feasibility and acceptability of its implementation.

NYKS, an autonomous organization under the Ministry of Sports and Youth Affairs, mobilizes young people for community development activities through a widespread network of local youth clubs, each comprising 10–15 volunteers (10).

### 1.1 Objective

1. To develop a logic model encompassing inputs, processes, outputs, and outcomes by collaborating with NYKS “youth club” members to engage them in the active screening and motivation of persons with TB-suggestive symptoms in the local community, and
2. To gain deeper insights into the feasibility, acceptability, challenges, and suggestions among stakeholders.

## 2 Methodology

### 2.1 Study design

A mixed-method study design was adopted to achieve the stated objectives. The logic model was developed using standard methodology reported earlier (11). A qualitative study was undertaken to assess the feasibility, acceptability, challenges, and suggestions for improvement.

### 2.2 Study setting

The study was carried out in six districts across five Indian states: Jaipur in Rajasthan, Lucknow in Uttar Pradesh, Patna in Bihar, Pune in Maharashtra, and Bhopal and Ratlam in Madhya Pradesh.

### 2.3 Sampling methodology

A multistage sampling technique was adopted. In the first stage, the six study districts were purposively selected as they represented the operational areas of the collaborating investigators and provided feasibility for multi-site fieldwork. All selected districts have an established NYKS presence and a substantial rural population where the TB burden and delays in care-seeking are known to be higher. In the second stage, within each district, two blocks with relatively higher TB burden were identified in consultation with district/state TB officers to ensure the inclusion of settings where the logic model activities were most relevant. Villages were randomly selected from the list of villages with active NYKS youth clubs, as the implementation of the proposed model required the presence of a functioning local volunteer platform. In the third stage, participants were purposively selected from the NYKS structure, including NYKS youth club members, block volunteers, district coordinators, health system personnel (including Medical Officers, Lab Technicians, DOTS providers, DBT managers, and nursing staff), and community leaders. The sampling was structured to ensure representation of all stakeholder groups involved in implementing or experiencing the proposed model, rather than to achieve statistical representativeness of any larger population.

### 2.4 Sample size

The sample size was determined by data saturation at each site.

### 2.5 Data collection methods and instruments

Data collection instruments were developed through a review of existing literature, expert consultations, and pilot testing, with subsequent revisions made to enhance clarity and ensure alignment with study objectives.

Developed instruments included five interview guides—three key informant interview (KII) guides for health system staff and NYKS officials, and two in-depth interview (IDI) guides for community leaders and village health workers. Additionally, two survey questionnaires profiling health system stakeholders and NYKS youth club members, and one focus group discussion (FGD) guide were developed.

### 2.6 Logic model development

A logic model represents the objectives, actions, and expected achievements of a program visually. The model, comprising four key domains—inputs, processes, outputs, and outcomes—establishes the logical connections between its resources, activities, and benefits (11). Its development involves defining the purpose and scope, identifying domains, drafting the model, refining it, and then finalizing it (11). Many authors have emphasized the importance of connecting activities, outputs, outcomes, and impacts (12, 13).

This logic model was also developed through a standard methodology that included: (1) a review of existing policies, reports, and scholarly literature, (2) seeking opinions from stakeholders, (3) drafting, and (4) refinement and finalization (11).

#### 2.6.1 Stage 1 (review of existing policies, reports, and scholarly literature)

Old and existing TB control policies of WHO and the Government of India were thoroughly reviewed. Current strategies, protocols, and recommendations for TB detection were emphasized. The overarching goals, priorities, and approaches to eliminate TB received attention. WHO guidelines on TB control and active case finding provided a broader perspective and best practices (14). Policies of the Government of India regarding primary healthcare, community engagement, and health system strengthening were analysed thoroughly (15).

A study focusing on TB control that discussed community engagement and successful case-finding models was also reviewed (16). The study captured the community and stakeholders’ perspectives, including an understanding of challenges and potential solutions for active case finding (17).

#### 2.6.2 Stage 2 (stakeholders’ assessment & opinion seeking)

An assessment of stakeholders was undertaken to understand the *Nehru Yuva Kendra Sangthan* (NYKS) system in depth. The organizational structure, operational processes, and overall functioning were examined meticulously. The NYKS leadership was discussed at all sites. The purpose of this discussion was to understand the roles and responsibilities of its members, the framework of community engagement, the workflow within the organization, the type and frequency of activities, and the level of engagement.

Additionally, the local healthcare system was explored for better understanding. Key program personnel in the public health system were consulted for their assessments and opinions on the proposed logic model. The mode of functioning, specific roles and responsibilities of healthcare professionals operating at the peripheral level, especially auxiliary nurse midwives (ANMs) and accredited social health activists (ASHAs), and the challenges they encounter were emphasized in the discussion.

#### 2.6.3 Stage 3 (development of draft logic model)

The logic model was drafted, outlining the required personnel, resources, and activities after stages 1 and 2. Monitoring indicators were formulated to assess progress effectively. It was drafted around four key domains: input, process, output, and outcome (11–13). Relevant activities designed for each domain included (1) information, education, and communication (IEC) activities, (2) screening, (3) referral, and (4) case detection. The model meticulously defined input, process, output, and outcome domains for each of the four activities. This systematic approach not only facilitates implementation and management but also enables the monitoring of the logic model to evaluate overall success.

#### 2.6.4 Stage 4 (Refinement and finalization of logic model)

Model refinement was undertaken iteratively after the initial draft. This phase comprised multiple rounds of stakeholder feedback and a comprehensive peer review by project investigators and coordinators. Valuable and diverse perspectives from stakeholders were meticulously collated and integrated into the model. The refined logic model was then discussed with stakeholders from NYKS and the public health system to finalize it. This study aimed to optimize the effectiveness and feasibility of the logic model, aligning it more closely with the dynamic needs and expectations of stakeholders and the target community.

### 2.7 Conceptual model of data collection instruments

The interview and FGD guides were developed to generate the evidence required for assessing the feasibility and acceptability of the proposed logic model for active TB screening. The questions were aligned with the core activities of the model (information, education, and communication (IEC), screening, referral, and case detection) and explored relevant constructs such as feasibility, acceptability, perceived enablers, barriers, and suggestions for improvement. Each guide contained open-ended, stakeholder-specific questions that enabled participants to comment on how the proposed activities fit within existing resources, processes, and expected outcomes. This design ensured that the instruments were conceptually linked to the logic model and adequately captured stakeholder insights necessary for examining its practical implementation.

### 2.8 Data collection procedures

Interviews and FGDs were conducted face-to-face by 12 trained researchers (7 male, 5 female) across all six study sites. Rapport was established in advance with village leaders, NYKS members, and healthcare providers, and participants were briefed on the study objectives. All interviews and FGDs were held in private spaces within participants’ localities to maintain confidentiality and minimize external disturbance. The local language was used throughout, supplemented by detailed field notes to capture contextual and non-verbal information. Open-ended surveys were administered to key informants to complement qualitative findings and enrich socio-demographic and organizational understanding. The combined use of KIIs, IDIs, FGDs, and surveys ensured triangulation of perspectives from community members, youth volunteers, and health system staff.

### 2.9 Data transcription and translation

Audio files of interviews and FGDs were thoroughly listened to multiple times to gain familiarity and clarity on the data. Audio data was then transcribed in the local language verbatim. Field notes were referred to clarify the audio data when needed and to capture non-verbal responses. Data collectors were also consulted during transcription to minimize data loss. These transcripts were reviewed multiple times before being translated into English for further analysis. Transcripts were systematically coded and indexed by participant ID and site location to ensure data traceability and rigor. All personal identifiers were removed during transcription, and only participant IDs were retained to ensure anonymity. Any names, locations, or contextually identifying details were excluded from both the transcripts and translated versions. Audio files and transcripts were stored in password-protected folders with access restricted to the research team, ensuring confidentiality throughout transcription, translation, and analysis.

### 2.10 Data analysis

The quantitative indicators referenced in the manuscript pertain only to the monitoring indicators defined within the logic model; no numerical data were collected for these indicators in this study, and they were not analysed statistically. The qualitative data analysis was conducted as described below:

- *Coding Framework*: Data were coded using a hybrid deductive–inductive approach. Deductive codes were based on the three predefined categories drawn from the study objectives and interview guide structure: reasons for acceptance, barriers, and suggestions for improvement. Inductive sub-codes were developed from concepts emerging directly from the transcripts within each category. Coding was carried out in NVivo, and coded datasets were reviewed by the research team to refine code definitions, ensure consistency, and resolve discrepancies.

To enhance coder reliability, coded datasets from all sites were cross-checked by the central analysis team; discrepancies were discussed and resolved collectively, and code definitions were refined iteratively. Triangulation was ensured through the inclusion of multiple stakeholder groups, multi-site data, and the combined use of interviews, FGDs, and surveys.

- *Thematic analysis*: Thematic Analysis was conducted with the primary objective of identifying key factors related to the feasibility and acceptability of the proposed logic model. Three broad code categories were predefined based on the study objectives and interview guide structure: (1) reasons for acceptance, (2) barriers, and (3) suggestions for improvement. All site teams coded their transcripts under these categories, and additional sub-codes were generated inductively from participant narratives within each category. This ensured that while the overarching structure remained consistent across sites, the finer codes reflected the actual experiences and perspectives emerging from the data.

Data from all sites were consolidated into a unified thematic structure. Convergence and divergence across sites were examined, and overlapping sub-codes were merged to produce final cross-site themes. Representative extracts from different participant groups were used to support each theme. This process enabled the synthesis of locally grounded findings into a coherent cross-site interpretation of feasibility, acceptability, facilitators, and barriers related to the logic model activities.

### 2.11 Ethical considerations

The study followed ethical guidelines issued by the Indian Council of Medical Research (ICMR) (18). The Institute Ethics Committee of ICMR – National Institute for Implementation Research on Non-Communicable Diseases, Jodhpur, evaluated the research protocol before granting ethics approval (File No. IEC-ICMR-NIIRNCD/2021/25/3). Written informed consent was obtained from all participants.

### 2.12 Reporting standard

The study adheres to the standard methodology of the logic model and the Consolidated Criteria for Reporting Qualitative Research (COREQ) checklist (11, 19).

## 3 Results

**Figure 1:**
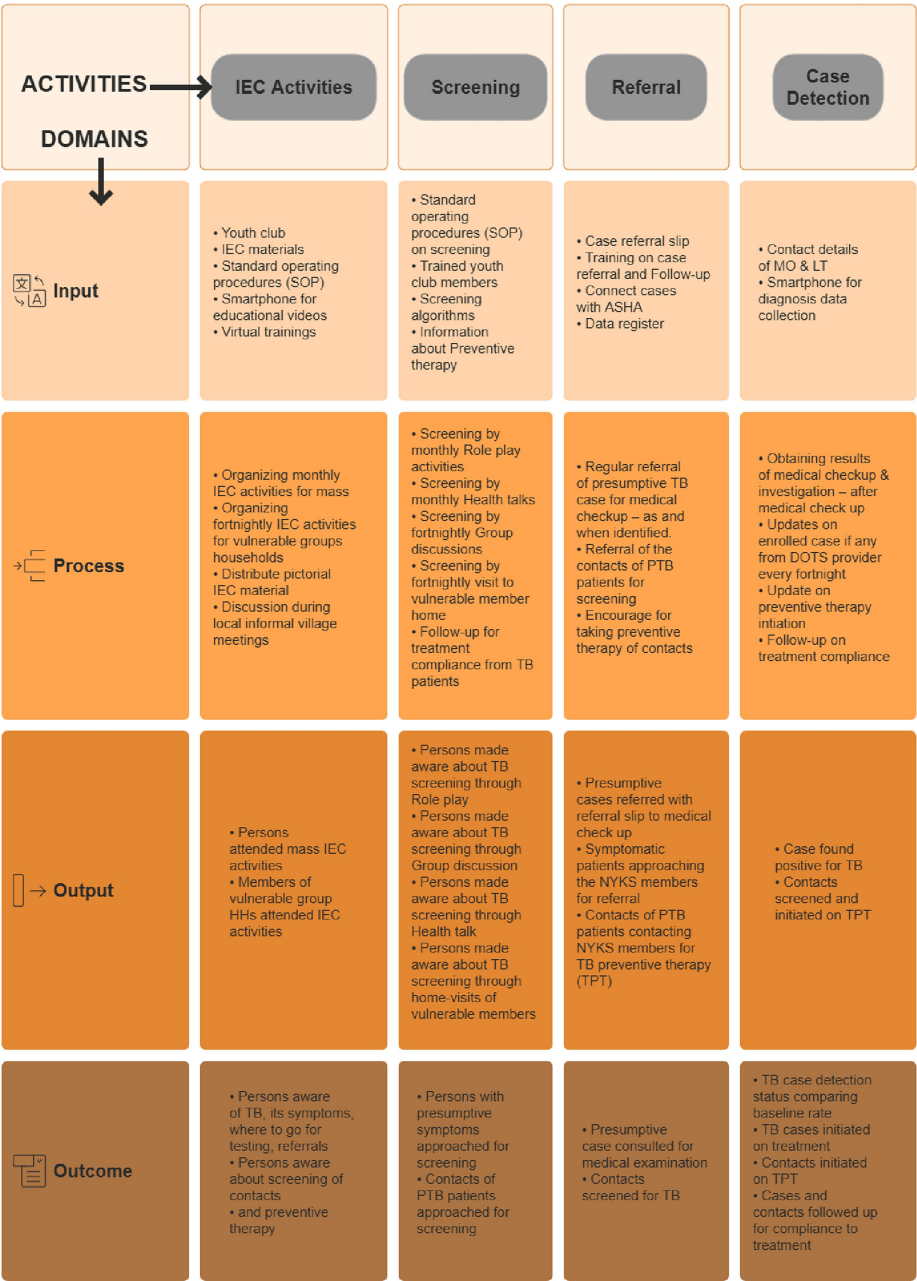
NYKS logic model.

**Figure 2:**
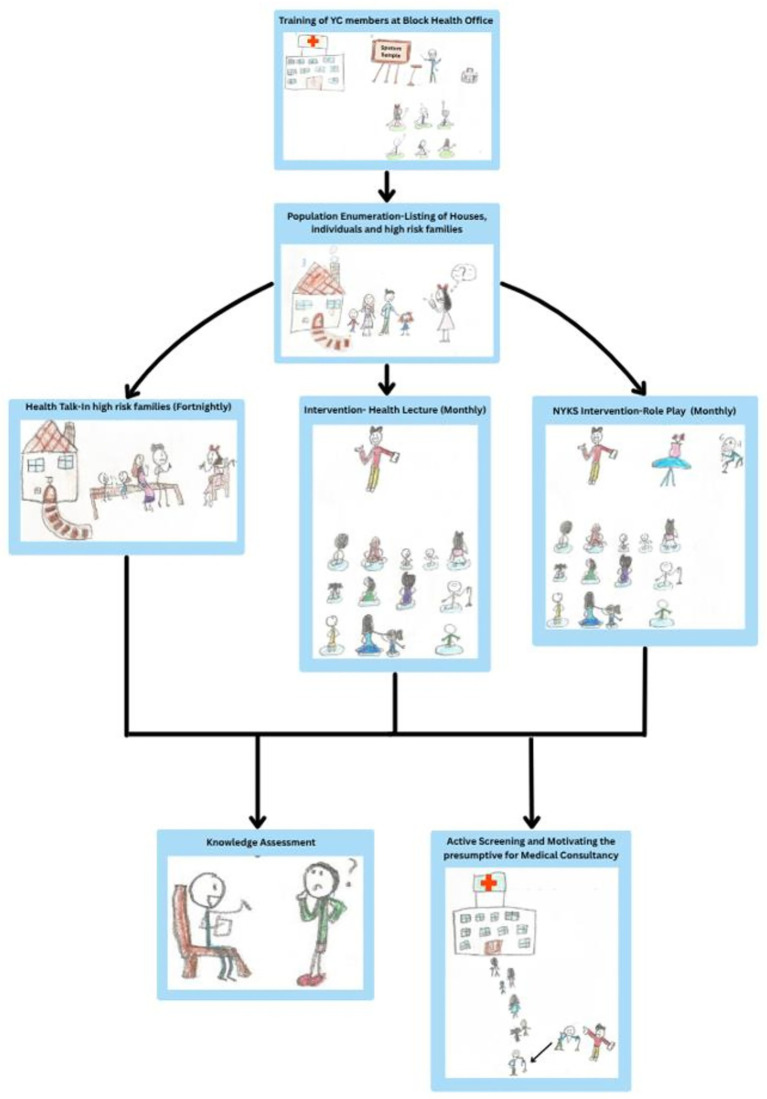
NYKS logic model schema of activities. [Drawing courtesy Ms. Aarna Anand (Chhota), Vth class, Delhi Public School, Pali Road, Jodhpur].

In Figure 1, a visual description of different domains and corresponding activities is presented within the “NYKS logic model”. For each included activity, input, process, output, and outcome domain indicators are meticulously outlined, creating a thorough framework for monitoring purposes. These indicators encompass a variety of factors, including resource allocation, activity execution, immediate outcomes, and the broader impact on the community. This structured approach facilitates a comprehensive assessment, enabling the monitoring of various facets crucial to the model’s success. Figure 2 represents the implementation schema of the NYKS logic model by trained volunteers from youth clubs. The schema demonstrates the practical workflow of the model, involving a one-day training for volunteers, population enumeration to identify high-risk families and the total population under the model, intervention activities, and presumptive case identification and referral, all for an easy understanding of the NYKS logic model.

The following activities were included in this logic model.

### 3.1 Information, education, and communication (IEC) activities

For information, education, and communication (IEC) activities, specific inputs were identified for the “Input” domain of the logic model. These inputs included members of the youth club, IEC materials, standard operating procedures (SOP), and the provision of smartphones for educational videos. Two distinct activities were established for the “Process” domain of the logic model. These IEC processes included performing activities targeting the general population on a monthly basis and another focusing on vulnerable groups (people living with diabetes and HIV/AIDS, contacts of PTB patients, people who work in mines or reside near mines, chronic cough patients, etc.) within households on a fortnightly basis. For the “Output” domain of IEC activities, output will be measured by quantifying the number of individuals attending IEC activities and the number of members from vulnerable group households participating in these sessions. These quantifiable outputs will help assess the overall outcome of these IEC activities. This outcome will be evaluated through the number of individuals made aware of tuberculosis transmission and diagnosis, as mentioned in the “Outcome” domain of IEC activities. To ensure effective oversight of IEC activities, key indicators have been defined across various domains. Table 1 describes the indicators that serve as benchmarks and metrics to systematically assess and monitor the performance and outcomes of IEC activities.

**Table 1:** Indicators to study for “IEC activities” and “screening” activities under “logic model”.

Domains	IEC activities	Screening
Input	No. (%) of clusters/villages with youth club No. (%) of youth club members with IEC materials viz. Standee, Banners, booklet, videos No. (%) of Youth club members with standard operating procedures (SOP) on conducting IEC activities No. (%) of youth club members with smart phones for showing educational videos	No. (%) of youth club members provided with standard operating procedures (SOP) on screening No. (%) of youth club members trained in screening No. (%) of members with screening algorithm
Process	No. (%) of village residents had received at least 1 IEC activity per month No. (%) of houses with vulnerable group members visited for “family discussion” every fortnight	No. of “role play” activities undertaken No. of “group discussion” held No. of “health talk” delivered No. of HHs visited to vulnerable member home
Output	No. (%) of individuals residing in cluster/village attended “Health talk” at for the month No. (%) of members of houses of vulnerable groups received “family discussion” for fortnight	No. of residents made aware about the TB screening through “role plays” No. of residents made aware about the TB screening through “group discussions” No. of residents made aware about the TB screening through “health talks” No. of residents made aware about the TB screening through vulnerable members household visits.
Outcome	No. (%) of persons found aware of TB after “health talk” No. (%) of members of vulnerable group HHs found aware of TB after “family discussion”	No. of persons with presumptive symptoms approached for screening No. of persons found with presumptive TB

Input domain indicators for IEC activities are (a) No. (%) of clusters/villages with youth clubs; (b) No. (%) of youth club members with IEC materials such as standee, banners, booklets, and videos; (c) No. (%) of youth club members with standard operating procedures (SOP) for conducting IEC activities; and (d) No. (%) of youth club members with smartphones for viewing educational videos. Process domain indicators for IEC activities are (a) No. (%) of village residents who received at least one IEC activity per month; and (b) No. (%) of households with vulnerable group members visited for “family discussions” every fortnight. Output domain indicators are (a) No. (%) of individuals in the cluster/village who attended a “Health talk” on a monthly basis; and (b) No. (%) of members of households with vulnerable groups who participated in “Family discussions” every fortnight. Outcome domain indicators for this activity are (a) No. (%) of persons aware of TB transmission and diagnosis after the “Health talk”; and (b) No. (%) of members of vulnerable group HHs who are aware of TB after the “Family discussion.”

### 3.2 Screening

Figure 1 illustrates the screening activity of this model. For the screening activities targeting presumptive tuberculosis (TB) patients, a strategic selection of inputs was determined under the “Input” domain of screening activities. This involves training youth club members on the screening process for presumptive cases, providing them with the standard operating procedures (SOP) for screening, and utilizing a screening algorithm to identify potential TB cases. Subsequently, four distinct activities were assigned, as mentioned under the “Process” domain of this screening activity: screening through monthly role play activities and health talks, and screening via fortnightly group discussions and household visits to vulnerable members. These activities aim to raise awareness about the symptoms indicative of presumptive TB cases, encouraging individuals to seek testing if such symptoms persist or if they are contacts of a PTB positive case. The outputs of these activities are gauged through metrics such as the number of people made aware of TB screening through the role plays, group discussions, health talks, and home visits to vulnerable members, as outlined under the “Output” domain. The “Outcome” domain of screening activities indicates that, by utilizing these outputs, the effectiveness of the screening domain can be assessed by how many individuals with presumptive symptoms approached for screening. To ensure effective oversight of screening activities, key indicators have been defined across various domains for this activity (Table 1).

Input domain indicators include (a) No. (%) of youth club members provided with standard operating procedures (SOP) on screening; (b) No. (%) of youth club members trained in screening; and (c) No. (%) of members with a screening algorithm. Process indicators are (a) No. of “Role play” activities undertaken; (b) No. of “Group discussions” held; (c) No. of “Health talks” delivered; and (d) No. of HHs visited to reach vulnerable members. Output indicators include (a) No. of residents made aware of TB screening through “Role plays”; (b) No. of residents made aware of TB screening through “Group discussions”; (c) No. of residents made aware of TB screening through “Health talks”; and (d) No. of residents made aware of TB screening through visits to vulnerable members’ households. Outcome indicators are (a) No. of persons with presumptive symptoms approached for screening; and (b) No. of persons found with presumptive TB.

### 3.3 Referral

In the referral activity for presumptive tuberculosis (TB) cases led by community volunteers, a systematic approach has been established (Figure 1). The initial step involves providing comprehensive training to these volunteers on the intricacies of the case referral process. This includes familiarizing them with the case referral slip and equipping them with a data register, as mentioned under the “input” domain of referral activities. Subsequently, as indicated in the “process” domain, the community volunteers are entrusted with the responsibility of referring presumptive TB cases for medical check-ups whenever necessary. The “output” domain of the referral activity shows that the output of this activity is quantified by the number of presumptive cases referred, accompanied by a referral slip, for medical check-ups. The cases can also be connected to ASHA for sputum collection and testing. The impact of this referral process is further evaluated by assessing the number of presumptive cases that ultimately consult for medical examinations or get sputum tested for TB, as mentioned under the “outcome” domain.

To ensure effective oversight of referral activities, key indicators have been defined across various domains. These indicators serve as benchmarks and metrics to assess and monitor the performance and outcomes of referral activities systematically (Table 2). Input domain indicators are (a) No. (%) of club members with a “Case referral slip”; (b) No. (%) of members provided training on case referral; and (c) No. (%) of club members provided with a data register. Process indicators are (a) No. (%) of club members referring presumptive cases for medical check-ups; and (b) No. (%) of club members maintaining records of referred presumptive cases in the register for follow-up. The output indicator is (a) No. (%) of referred presumptive cases with a referral slip that reached for medical check-ups. The outcome indicator is (a) No. (%) of referred presumptive cases examined by the MO for TB.

**Table 2:** Indicators to study for “referral” and “case detection” activities under “logic model”.

Domains	Referral	Case detection
Input	No. (%) of club members with “case referral slip” No. (%) of members provided training on case referral No. (%) of club members provided with data register	No. (%) of youth club members provided with contact details of MO & LT No (%) of club members with access to smartphone for diagnosis data collection & transmission
Process	No. (%) of club members referring the presumptive case for medical check up No. (%) of club members maintaining the record of referred presumptive cases in register for follow up	No. (%) of youth club members obtaining results of medical checkup & lab examination No. (%) of youth club members receiving updates on enrolled case if any from DOTS provider
Output	No. (%) of referred presumptive cases with referral slip reached to medical check up	No. (%) of presumptive cases found positive for TB
Outcome	No. (%) of referred presumptive cases examined by MO for TB	TB case notification rate in no. of cases notified/100,000 population for the area

### 3.4 Case detection

In the case detection phase of this tuberculosis (TB) intervention, volunteers who have access to smartphones for diagnostic data collection will be provided with the contact details of the medical officer (mo) and laboratory technician (LT) as mentioned in the “input” domain of the case detection activity (Figure 1). Subsequently, as indicated under the “process” domain of this case detection phase, the primary responsibility of these volunteers involves obtaining the results of medical check-ups/investigations and collecting updates on enrolled cases from the Directly Observed Treatment, Short-course (DOTS) provider on a fortnightly basis. The output of this activity will be determined by the total number of cases identified as positive for TB, as mentioned under the “output” domain section. The “outcome” domain indicates that the broader impact of this case detection activity can be evaluated by comparing the TB case detection status against the baseline rate.

Case detection activity indicators have been defined across various domains in Table 2. Input domain indicators are (a) No. (%) of youth club members provided with contact details of the MO & LT; and (b) No. (%) of club members with access to smartphones for diagnostic data collection & transmission. Process indicators are (a) No. (%) of youth club members obtaining results of medical check-ups & lab examinations; and (b) No. (%) of youth club members receiving updates on enrolled cases, if any, from the DOTS provider. The output indicator is (a) No. (%) of presumptive cases found positive for TB. The outcome indicator is (a) TB case notification rate in No. of cases notified/100,000 population for the area.

## 4 In-depth qualitative analysis

This study explored the feasibility and acceptability of engaging NYKS “youth club” members in the active screening and motivation of presumptive TB cases, as well as their effectiveness and barriers. Using thematic analysis, the study identified key patterns related to the model’s acceptance, feasibility, barriers, and suggestions for improvement.

The findings also offer important insights into the sustainability and operational viability of a NYKS-led TB screening model. The study was conducted across six districts from five states of India, involving 414 participants (67% male). Detailed participant demographics are presented in Table 3.

**Table 3:** Participant characteristics.

State name	Site name	Stakeholder category	No. of participants		
			Male	Female	Total
Rajasthan	Jaipur	Nehru Yuva Kendra Sangthan	32	0	32
		Public Health	8	7	15
		Community	5	6	11
Uttar Pradesh	Lucknow	Nehru Yuva Kendra Sangthan	15	7	22
		Public Health	3	4	7
		Community	4	1	5
Maharashtra	Pune	Nehru Yuva Kendra Sangthan	15	0	15
		Public Health	5	4	9
		Community	1	0	1
Bihar	Patna	Nehru Yuva Kendra Sangthan	48	5	53
		Public Health	1	6	7
		Community	2	5	7
Madhya Pradesh	Bhopal	Nehru Yuva Kendra Sangthan	43	13	56
		Public Health	8	38	46
		Community	20	8	28
	Ratlam	Nehru Yuva Kendra Sangthan	51	5	56
		Public Health	11	23	34
		Community	7	3	10

### 4.1 Acceptance of the model

**Figure 3:**
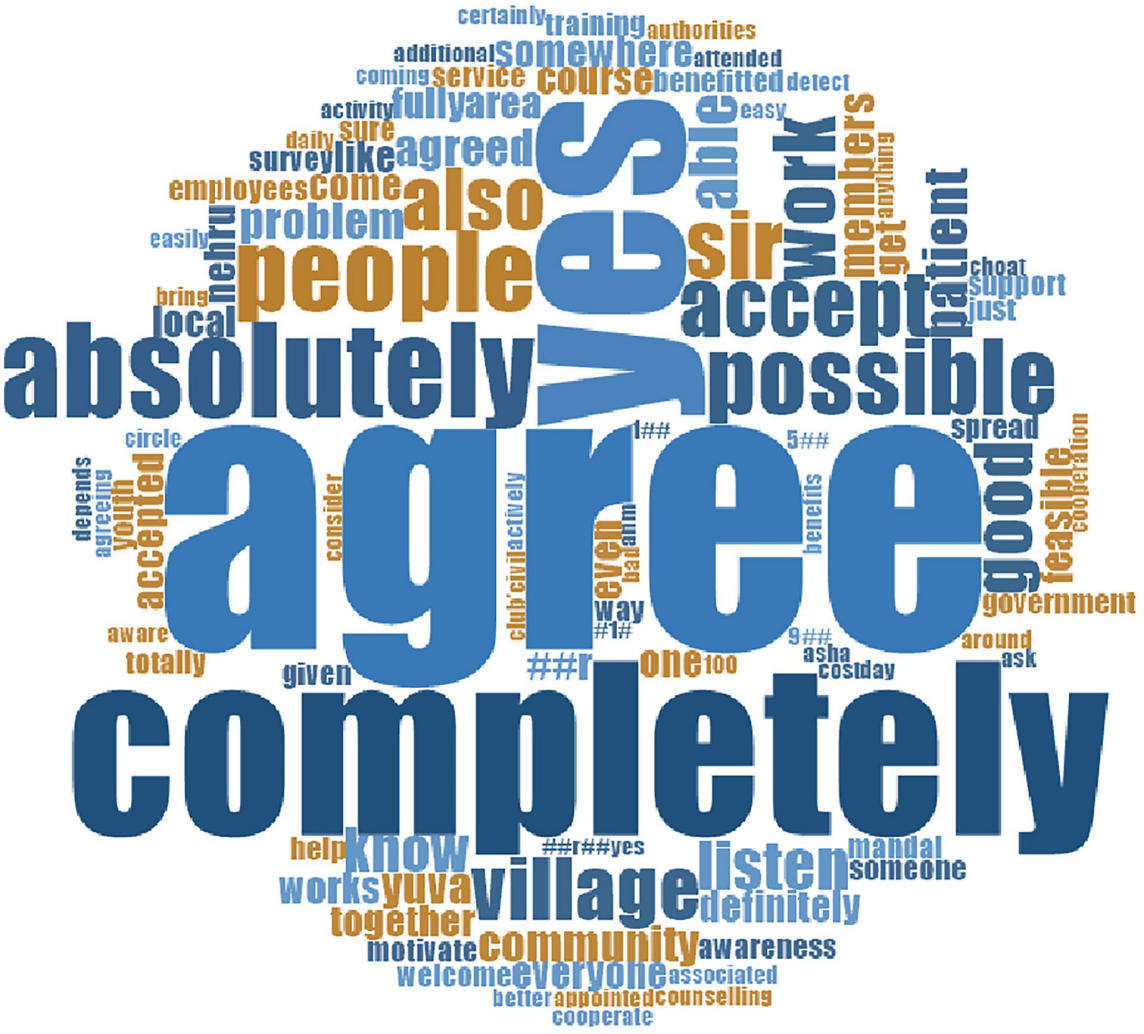
Acceptance of model.

The model received broad acceptance from most participants, among five distinct categories of responses, including (1) complete acceptance, (2) conditional acceptance, (3) tentative disagreement, (4) strong disagreement, and (5) uncertainty (Figure 3).

#### 4.1.1 Complete acceptance

Participants from all stakeholder groups widely endorsed the model. Their support was rooted in the belief that local youth volunteers were well-positioned to engage communities due to shared identity and trust. Participants emphasized that youth are effective communicators and acknowledged the need for a collective effort. These views collectively underscored the perceived feasibility and value of the model in enhancing TB awareness and early detection (Table 4, Q1–Q4).

**Table 4:** Quotes related to acceptance of the model and reason for acceptance.

Sr. no.	Quote (respondent, site)	Interpretation	Key concept	Theme
Q1.	“People will support them as they are their community members.” (Doctor, Pune)	Local volunteers are trusted due to familiarity with community.	Shared identity, Trust	Complete acceptance
Q2.	“If youth of our country come forward, they can motivate everyone.” (Health worker, Bhopal)	Youth are seen as effective motivators for social causes.	Youth agency, Social influence	
Q3.	“These villagers will not listen to the ASHA, ANM and CHO at all… if everyone works together, it is possible.” (ANM, Jaipur)	Collective effort is viewed as necessary for success.	Collective action, collaboration	
Q4.	“I completely agree, it’s very good, people often do not even realize they have TB.” (Community Representative, Bhopal)	Endorses model as a way to increase TB awareness.	Health awareness, model feasibility	
Q5.	“We will certainly do it if we receive order from senior authorities.” (Doctor, Pune)	Support is conditional upon formal approval from higher-level authorities.	Hierarchical accountability, top-down governance	Conditional acceptance
Q6.	“We do not have any problem if someone gets benefitted.” (ANM, Jaipur)	Indicates neutral stance, model acceptable on observable benefits to the community.	Pragmatism, community-oriented flexibility	
Q7.	“They will also listen to them… but they listen to us more.” (ANM, Jaipur)	Acknowledges NYKS influence, but places health workers higher in the trust hierarchy.	Relative trust, role authority	
Q8.	“If it’s in the village, we will be able to do all these things.” (NYKS Member, Patna)	Feasibility depends on location; community-level implementation seen as manageable.	Local feasibility, operational clarity	
Q9.	“Only symptomatic will answer… normal people will not.” (NYKS, Bhopal)	Suggests doubts about the reach and engagement potential of the model.	Perceived limitations of community screening	Tentative disagreement/doubt
Q10.	“ASHA workers are already given priority.” (Doctor, Ratlam)	Indicates skepticism about the model’s added value vis-à-vis existing systems.	Redundancy with current roles/trust hierarchy	
Q11.	“There are already so many people in health services… there’s no need to involve new youth.” (Lab Technician, Bhopal)	Views youth involvement as unnecessary due to existing staff availability.	Redundancy concerns/perceived workforce sufficiency	Strong disagreement
Q12.	“We already have dedicated workers like Anganwadi and health workers… they know things NYKS might not.” (NYKS Member, Bhopal)	Suggests current personnel are better informed and more trustworthy.	Trust in existing workforce/role competence	
Q13.	“How can I agree right now when I do not even know about it?… I give my agreement after getting full information.” (STS, Bhopal)	Participant expressed openness but emphasized the need for adequate information prior to commitment.	Incomplete information, conditional openness, need for clear communication	Uncertainty
Q14.	“People will trust them because they are local people, they have belief in him.” (ANM, Pune)	Trust arises from local embeddedness	Trust in local volunteers	Local implementation unit
Q15.	“People in village do not disclose their clinical status to unknown persons… myself being local, I know who is having what problems.” (NYKS Member, Bhopal)	Community members disclose more to insiders	Local trust & familiarity	
Q16.	“They will listen to us 10%, they will listen to them 70%.” (Doctor, Pune)	Volunteers have stronger influence than health workers	Preference over formal staff	
Q17.	“It will be good… they know about their village.” (ANM, Jaipur)	Familiarity enhances rapport	Recognition & acceptance	
Q18.	“The one from the same village will do counselling well… will know whose health is bad.” (Doctor, Jaipur)	Local knowledge improves counselling	Local understanding	
Q19.	“If youth club members are from the same village… awareness and screening will be easy.” (Doctor, Ratlam)	Shared identity facilitates communication	Enhanced communication	
Q20.	“Yes, completely agree… villagers will not tell us, but will tell them.” (ANM, Jaipur)	NYKS members more approachable	Preference for community peers	
Q21.	“If services are available to us, we will not wander… patient will get proper treatment.” (Doctor, Bhopal)	Local access improves service uptake	Easy accessibility	
Q22.	“Community cooperation is necessary… if all work together, it is possible.” (DOTS Provider, Ratlam)	Local synergy supports implementation	Community support	
Q23.	“Yes… if all work together, then only India will be TB free… ASHA, ANM and CHO; villagers listen very little.” (DOTS Provider, Ratlam)	Existing staff have limited impact alone	Preference for local collaboratives	
Q24.	“I completely agree, if people come to know how TB can be treated, how one can fight TB, then everyone will take precautions, it’s good to provide information.” (Community leader, Bhopal)	Participants valued the role of the model in improving TB-related health literacy and promoting preventive actions.	Positive impact on health awareness	TB and health priority
Q25.	“It will be good. They come to village, raise awareness, there will be good publicity.” (ANM, Bhopal)	Awareness activities by NYKS were appreciated for encouraging community-level engagement and knowledge-sharing.	Support for health initiatives	
Q26.	“We must welcome that NYKS group… they should be support. Not just by Nehru Youth Club, but also by other groups who we have seen, there are many such organizations that encourage people, tell symptoms, raise awareness… I agree that symptomatic persons will be identified.” (Doctor, Bhopal)	The respondent expressed general support for all health initiatives that enhance awareness and case identification.	Support for health initiatives	
Q27.	“Because by detecting new cases of tuberculosis (TB) by youth club members, the treatment and examination of tuberculosis patient will be completed on time and the spread of (TB) Tuberculosis can be prevented.” (Doctor, Ratlam)	Early detection by local youth was seen as critical to breaking the chain of transmission and ensuring timely care.	Community well-being and TB management	
Q28.	“Madam, why would not I agree? This will improve our village; the people of our village will stay safe because they will have full information. These germs spreading will stop spreading, they will not spread further, then they will be completely eradicated.” (ANM, Bhopal)	The intervention was linked to broader health improvement and TB eradication at the community level.	Community well-being and TB management	
Q29.	“Completely agree, the government is conducting these free camps, if there are [disease], it can be detected in time and treatment can be given, there will be no problem.” (Community leader, Bhopal)	Support for preventive health programs was driven by the promise of early disease detection and timely treatment.	Local participation for effective TB control	
Q30.	“Agree will definitely welcome such type of group if recruited because we understand that ASHA are already overloaded…” (Healthcare Professional, Patna)	Participants welcomed the model as a means to reduce pressure on overstretched health workers by expanding staff capacity.	Increased staff of local healthcare system	Strengthening local healthcare system
Q31.	“Because by detecting new cases… the spread of TB can be prevented.” (Doctor, Ratlam)	Early case identification by NYKS members was viewed as direct support to TB screening and ACF efforts.	Activity support of local healthcare system	
Q32.	“It’s a public service. Social service work. No problem will come.” (NYKS Member, Jaipur)	The model’s social service orientation increased its acceptability, being seen as a moral and civic contribution.	Support for collective good	Social service acceptance

#### 4.1.2 Conditional acceptance

Conditional acceptance was voiced primarily by health workers across Jaipur, Patna, and Pune. This group indicated general agreement with the model’s goals but emphasized that participation was contingent upon formal directives or specific assurances. Some participants acknowledged the local influence of NYKS members while reinforcing the primacy of health staff within the community trust hierarchy. Others expressed logistical openness, highlighting that proximity and clarity would influence feasibility. Collectively, this group supported the model’s intent but stressed the need for better alignment with existing systems and clearly communicated mutual benefits (Table 4, Q5–Q8).

#### 4.1.3 Tentative disagreement/doubt

Some NYKS members (Bhopal) and healthcare staff (Ratlam) voiced hesitation regarding the model’s effectiveness, citing concerns about limited community responsiveness and potential redundancy with existing roles. Their responses reflected uncertainty about the added value of the model in its current form (Table 4, Q9–Q10).

#### 4.1.4 Strong disagreement

A small group from Bhopal and Ratlam viewed the initiative as redundant, given existing personnel and infrastructure. They advocated for strengthening current workers rather than introducing new personnel (Table 4, Q11–Q12).

#### 4.1.5 Uncertainty

Some participants, particularly from Pune and Bhopal, expressed a need for more information before committing. These respondents were open to supporting the model but needed clearer communication (Table 4, Q13).

### 4.2 Reason for acceptance

**Figure 4:**
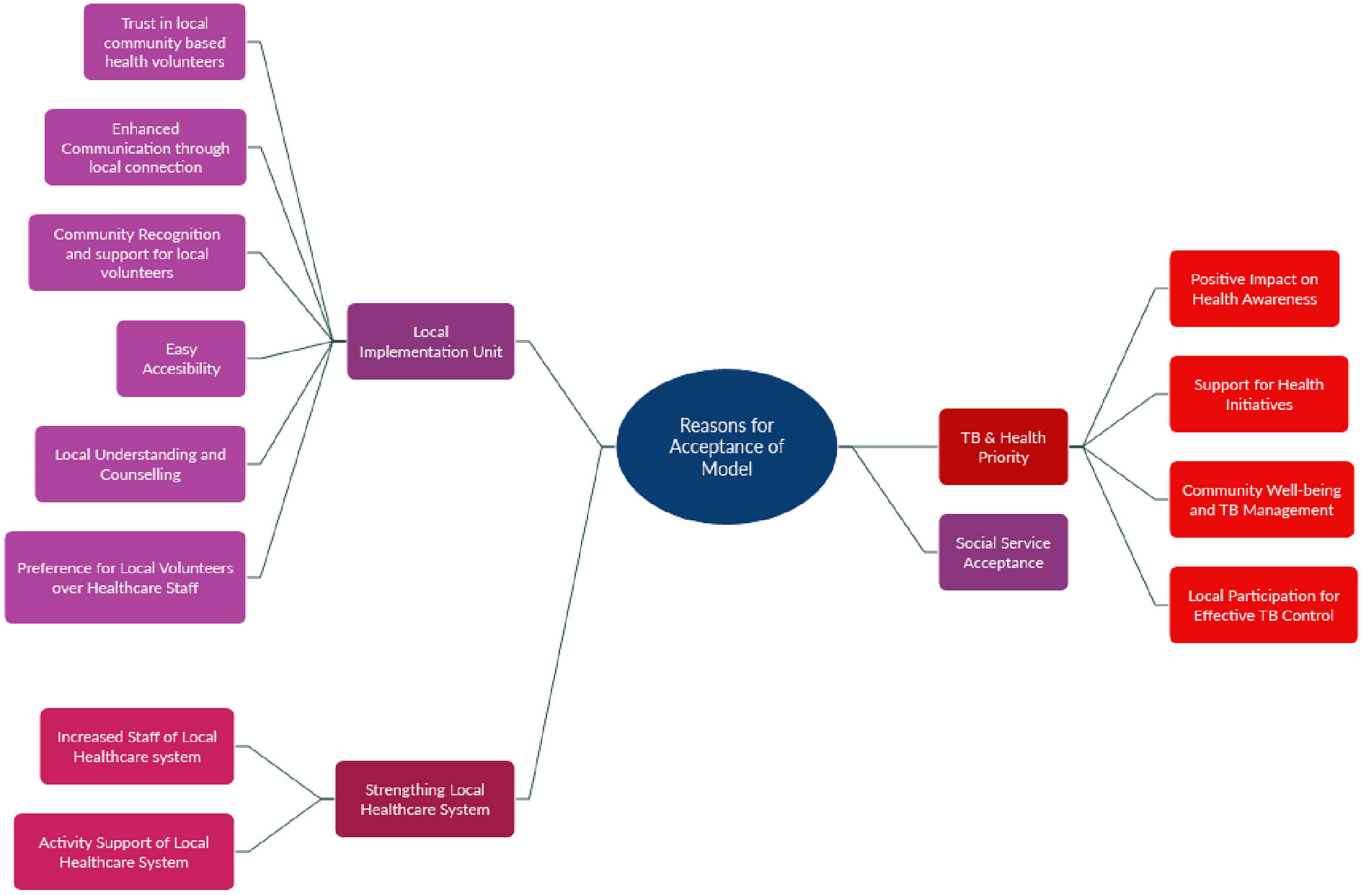
Reasons for acceptance of model.

The reasons for model acceptance under *Complete Acceptance* or *Partial Acceptance* categories are summarized in Figure 4. These reasons are structured into four key themes: (1) local implementation unit; (2) TB and health priority; (3) strengthening the local healthcare system; and (4) social service acceptance.

#### 4.2.1 Local implementation unit

A prominent reason for acceptance across study sites (Ratlam, Pune, Bhopal, and Jaipur) was the presence of a local implementation unit, specifically the NYKS volunteers. Participants emphasized the unique advantage of leveraging local youth who are embedded within the community. Their familiarity, trust, and existing social relationships were seen as critical to the acceptability and effectiveness of the intervention (Table 4, Q14–Q23).

##### 4.2.1.1 Trust in local community-based health volunteers

Community members expressed a high degree of trust in NYKS volunteers, facilitating open communication and participation in health initiatives.

##### 4.2.1.2 Enhanced communication through local connections

Shared language and cultural familiarity allowed NYKS volunteers to communicate health messages more effectively.

##### 4.2.1.3 Community recognition and support for local volunteers

NYKS members were viewed with respect and support, fostering a collaborative environment essential for implementation success.

##### 4.2.1.4 Easy accessibility

Respondents agreed that local NYKS members ensure timely access to health services by reducing delays in diagnosis and treatment.

##### 4.2.1.5 Local understanding and counselling

Volunteers’ awareness of local health needs enabled them to provide personalized health counselling and support.

##### 4.2.1.6 Preference for local volunteers over healthcare staff

Participants expressed a preference for local volunteers over professionals like ASHA, ANM, or CHO, citing greater comfort and trust with community-based peers.

#### 4.2.2 Tb and health priority

Acceptance was also driven by its alignment with health concerns, particularly TB. This theme was notably prominent among healthcare participants from Ratlam, Patna, Jaipur, and Bhopal (Table 4, Q24–Q29).

##### 4.2.2.1 Positive impact on health awareness

The intervention was expected to enhance awareness of TB.

##### 4.2.2.2 Support for health initiatives

There was broad endorsement for any initiative that supports community health through awareness and engagement.

##### 4.2.2.3 Community wellbeing and TB management

Addressing TB was considered vital for improving public health.

##### 4.2.2.4 Local participation for effective TB control

The involvement of local youth was seen as key to promptly identifying and managing TB cases, due to their proximity to the community.

#### 4.2.3 Strengthening local healthcare system

Healthcare professionals across sites acknowledged that the model would significantly contribute to strengthening the local healthcare system.

##### 4.2.3.1 Increased staffing in the local healthcare system

Participants, especially from Pune, Patna, and Bhopal, highlighted the overburdened status of frontline workers. The addition of trained NYKS volunteers was viewed as a valuable supplement (Table 4, Q30).

##### 4.2.3.2 Support activities for the local healthcare system

Respondents emphasized the role of NYKS volunteers in supporting active case finding (ACF). Volunteers were considered instrumental in early TB detection, conducting house-to-house screenings, and referring missed or undiagnosed cases (Table 4, Q31).

#### 4.2.4 Social service acceptance

Some participants (especially from Pune, Jaipur, and Bhopal) accepted the model on the grounds that it represented a form of social service. They viewed the initiative as a selfless, community-oriented intervention with intrinsic value (Table 4, Q32).

##### 4.2.4.1 Community motivation

Respondents appreciated the voluntary nature of the model and the willingness of youth to serve their communities, often at personal risk.

##### 4.2.4.2 Support for the collective good

The model was seen as aligned with social welfare and public service goals, enhancing its acceptability and perceived legitimacy.

### 4.3 Perceived barriers in the NYKS model

**Figure 5:**
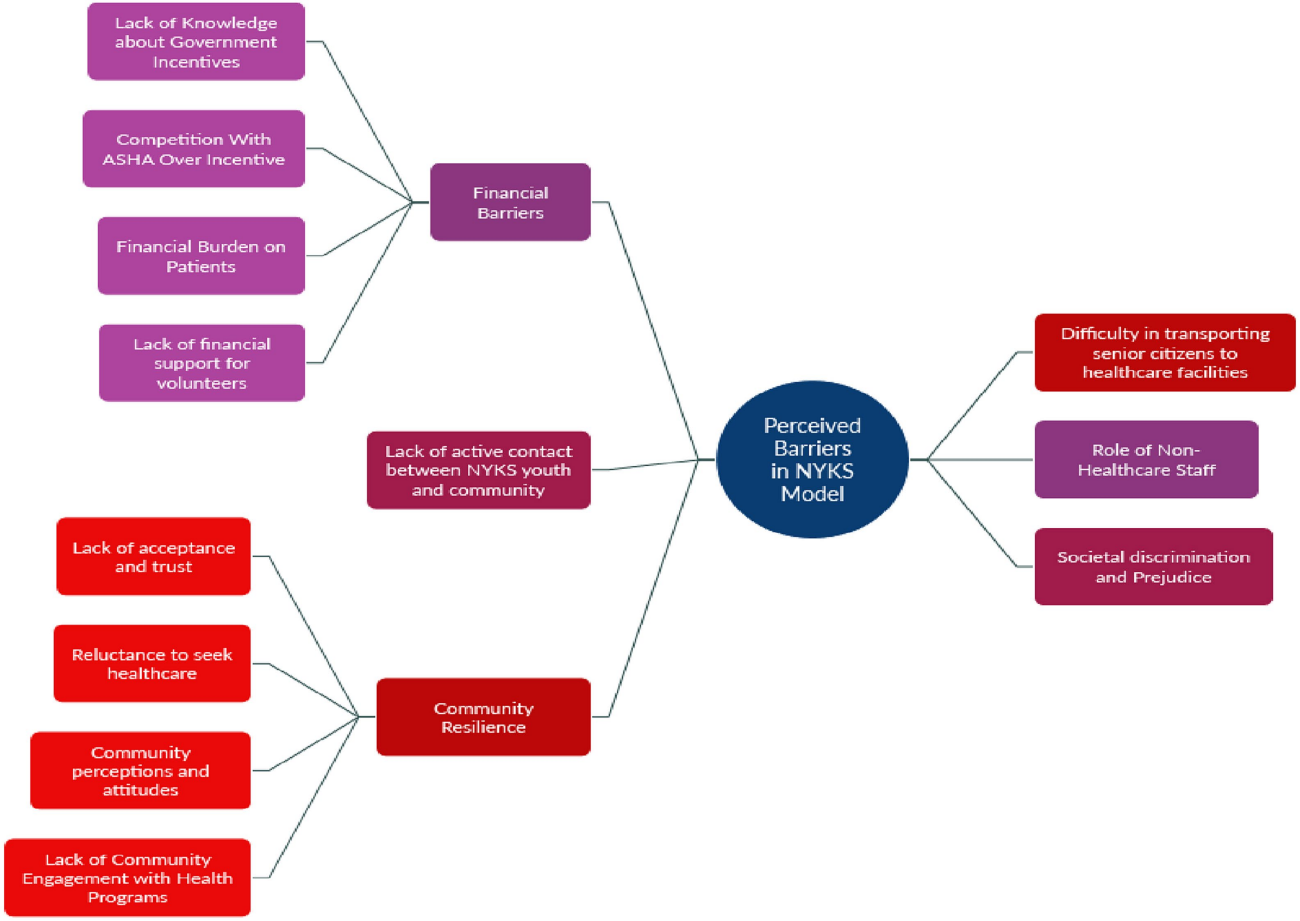
Perceived barriers in NYKS model.

Based on the analysis, several key challenges emerged (Figure 5).

#### 4.3.1 Financial barrier

##### 4.3.1.1 Lack of knowledge about government incentives

Participants from Pune, Patna, Jaipur, Bhopal, and Lucknow highlighted a significant lack of awareness regarding existing government schemes and incentives for volunteers involved in TB identification. Respondents expressed doubts about the availability and feasibility of providing incentives, indicating misunderstandings or lack of clarity regarding government support (Table 5, Q1–Q5).

**Table 5:** Quotes related to perceived barriers of NYKS model.

Sr. no.	Quote (respondent, site)	Interpretation	Key concept	Theme
Q1.	“There is no system of incentive but it should be given. It is important to increase effectiveness of work.” (ANM, Pune)	Incentives seen as necessary, but currently absent.	Lack of knowledge about government incentives	Financial barriers
Q2.	“Not possible at all. For DBT (Direct Bank Transfer)… It is not given to the common people.” (STS, Lucknow)	Misunderstanding DBT as only for patients, not workers.	Lack of knowledge about government incentives	
Q3.	“No, they do not get incentive, there’s no money involved, do not have information.” (ANM, Bhopal)	Complete lack of awareness about incentive systems.	Lack of knowledge about government incentives	
Q4.	“Not at all feasible… No, it will not be possible, they will not be given any kind of honorarium…” (Lab Tech, Jaipur)	Strong scepticism about government providing honorarium.	Lack of knowledge about government incentives	
Q5.	“Not feasible as of now, if in future any such scheme will come, I do not know about that.” (Doctor, Jaipur)	Ambiguity about future policies hinders planning.	Lack of knowledge about government incentives	
Q6.	“Presently ASHA, Anganwadi worker used to get this money but what will she think when these people will start getting this money directly?” (NYKS Member, Jaipur)	NYKS incentives could cause friction with ASHA workers.	Competition with ASHA over incentive	
Q7.	“Although it (providing informant incentive to NYKS member) cannot be done completely, because ASHA workers are already there.” (STS, Ratlam)	Existing health staff seen as already filling the role.	Competition with ASHA over incentive	
Q8.	“Sample testing targets are given to staff… Otherwise conflict for incentive may be there.” (Doctor, Pune)	Incentive-linked targets may clash between groups.	Competition with ASHA over incentive	
Q9.	“Patients do not reach here who live in urban slums… they have financial problems.” (STS, Bhopal)	Travel and access costs prevent care-seeking.	Financial burden on patients	
Q10.	“But still if he goes (/hospital/) for 5–6 days… cost of commuting… How will his house operate?” (NYKS Member, Jaipur)	TB treatment disrupts household income and logistics.	Financial burden on patients	
Q11.	“The main thing is that it will also cost, Sir… Who will pay for that?” (NYKS Member, Jaipur)	Cost concerns discourage volunteer participation.	Lack of financial support for volunteers	
Q12.	“If they do not receive financial support, they cannot do it.” (NYKS Member, Bhopal)	Without funding, youth cannot engage in sustained work.	Lack of financial support for volunteers	
Q12.	“If they will work, then they want money first, these days no one works for free.” (ANM, Jaipur)	General expectation of financial reward for any effort.	Lack of financial support for volunteers	
Q14.	“They do not believe blindly. Ask directly: What will we get? What will we benefit?” (NYKS Member, Jaipur)	Volunteers demand clear personal benefit before engaging.	Lack of financial support for volunteers	
Q15.	“If someone is investing their time… they deserve respect and should also receive money…” (LT, Bhopal)	Time investment seen as warranting financial recognition.	Lack of financial support for volunteers	
Q16.	“To send them to the hospital… like old parents lives here… they will not go by themselves… they will have to be taken along… there will be issues.” (NYKS Member, Jaipur)	Difficulty in transporting dependent elders may reduce the model’s practical utility for high-need groups.	Transport challenges for senior citizens	Difficulty in transporting senior citizens to healthcare facilities
Q17.	“All your plans are good, but the members of Nehru Yuva Kendra Sangathan who will do the work, where are they? They are not even in contact with us.” (Community Leader, Jaipur)	Lack of visibility and ongoing contact undermines trust and model credibility.	Weak community linkages	Lack of active contact between NYKS youth and community
Q18.	“There are many organizations run by the government… I do not consider all this good because we already have dedicated workers… like Anganwadi workers and health workers.” (NYKS Member, Bhopal)	Redundancy concerns due to already existing cadres of trained staff.	Perceived redundancy of NYKS Role	Role of non-healthcare staff
Q19.	“No need to enrol any new persons because there are already so many people in health services… Whether it’s a metro city or a village… all facilities are available.” (LT, Bhopal)	Questions on necessity of adding new personnel where systems already exist.	Preference for existing health workforce	
Q20.	“The villagers raise objections… What they (NYKS volunteers) will understand… what if they deliberately diagnosed someone with an illness.” (ANM, Bhopal)	Doubts about non-health staff’s ability and ethical competence in clinical settings.	Lack of trust in untrained volunteers	
Q21.	“Even if they have a cough or cold, they will still say they do not have anything… They will not allow anyone to come like that.” (NYKS Member, Bhopal)	Denial and mistrust hinder volunteers from identifying symptomatic individuals.	Lack of acceptance and trust	Community resilience
Q22.	“There is a bit of apprehension in the village… that people will come and deliberately diagnose us.” (Community Leader, Bhopal)	Fear of misdiagnosis leads to scepticism toward outreach efforts.	Lack of acceptance and trust	
Q23.	“Mostly people would not come there, or they would try to avoid.” (NYKS Member, Pune)	Individuals avoid care due to fear or stigma, reducing program reach.	Reluctance to seek healthcare	
Q24.	“Many people keep it hidden, do not tell that they have TB.” (Community Leader, Jaipur)	Concealment of illness undermines early detection and timely treatment.	Reluctance to seek healthcare	
Q25.	“People still fear TB… they do not go to areas where there are TB labs or wards… society looks at it with disdain.” (STS, Bhopal)	TB-related stigma deters individuals from accessing diagnosis or care.	Reluctance to seek healthcare	
Q26.	“Some slum dwellers will not agree… They belong to Bhopa caste… There may be problems with them.” (DOTS Provider, Jaipur)	Caste-linked perceptions fuel resistance toward health workers.	Community perceptions and attitudes	
Q27.	“These villagers will not listen to the ASHA, ANM and CHO at all.” (ANM, Jaipur)	Disregard for local health staff reflects deep-rooted distrust.	Lack of community engagement	
Q28.	“If we go by ourselves… people will not cooperate.” (NYKS Member, Bhopal)	Lack of rapport reduces volunteers’ effectiveness in community outreach.	Lack of community engagement	
Q29.	“There are many who do not even believe us.” (ANM, Jaipur)	Poor credibility and trust obstruct health communication.	Lack of community engagement	
Q30.	“There are many things like untouchability… we cannot express it fully… There are a lot of problems.” (DOTS Provider, Bhopal)	Untouchability-related practices limit access and acceptance of services.	Caste-based discrimination	Societal discrimination and prejudice
Q31.	“There may still be some discrimination in practice… like with the Harijans… These days everyone is intelligent… little bit is still in practice.” (DOTS Provider, Jaipur)	Persisting caste bias weakens inclusive healthcare delivery.	Caste-based discrimination	

##### 4.3.1.2 Competition with ASHA for incentives

Concerns were raised regarding perceived competition between NYKS volunteers and ASHA and Anganwadi workers over financial incentives. Participants from Ratlam, Pune, and Jaipur noted that introducing incentives for NYKS members might create conflict or demotivate existing staff due to overlapping responsibilities (Table 5, Q6–Q8).

##### 4.3.1.3 Financial burden on patients

Participants from Patna, Jaipur, and Bhopal reported that patients, particularly from economically disadvantaged backgrounds, face financial challenges related to transport, treatment access, and indirect costs, which may affect the reach and impact of the NYKS model (Table 5, Q9–Q10).

##### 4.3.1.4 Lack of financial support for volunteers

Participants across Ratlam, Patna, Pune, and Bhopal emphasized that NYKS members are unlikely to engage actively without financial support. There were repeated concerns about personal costs and expectations for compensation, with many asserting that volunteer work without incentives is unsustainable (Table 5, Q11–Q15).

#### 4.3.2 Difficulty in transporting senior citizens to healthcare facilities

A NYKS member from Jaipur pointed out the specific challenge of helping immobile senior citizens access healthcare (Table 5, Q16).

#### 4.3.3 Lack of active contact between NYKS youth and the community

The lack of regular engagement and visibility of NYKS members at the village level was identified as a potential obstacle. Without this, trust-building and effective communication are hindered (Table 5, Q17).

#### 4.3.4 Non-healthcare staff

Participants from Ratlam and Bhopal expressed concerns about the limited healthcare training of NYKS members. The necessity of involving non-healthcare youth was questioned, particularly in areas where trained government health personnel are already present (Table 5, Q18–Q20).

#### 4.3.5 Community resilience

##### 4.3.5.1 Lack of acceptance and trust

Scepticism, denial, and reluctance to share health information were reported as barriers, linked to poor awareness and misconceptions (Table 5, Q21–Q22).

##### 4.3.5.2 Reluctance to seek healthcare

Fear of stigma associated with TB, discourages individuals from accessing services or disclosing symptoms (Table 5, Q23–Q25).

##### 4.3.5.3 Community perceptions and attitudes

Negative attitudes towards healthcare workers were reported, particularly among marginalized and less educated groups (Table 5, Q26).

##### 4.3.5.4 Lack of community engagement with health programs

Participants reported limited trust and cooperation from community members, hindering outreach (Table 5, Q27–Q29).

#### 4.3.6 Societal discrimination and prejudice

Instances of caste-based discrimination were reported by health staff in Jaipur and Bhopal. These discriminatory practices can deter marginalized communities from engaging with healthcare services or NYKS volunteers (Table 5, Q30–Q31).

### 4.4 Suggestions for successful implementation of the model

**Figure 6:**
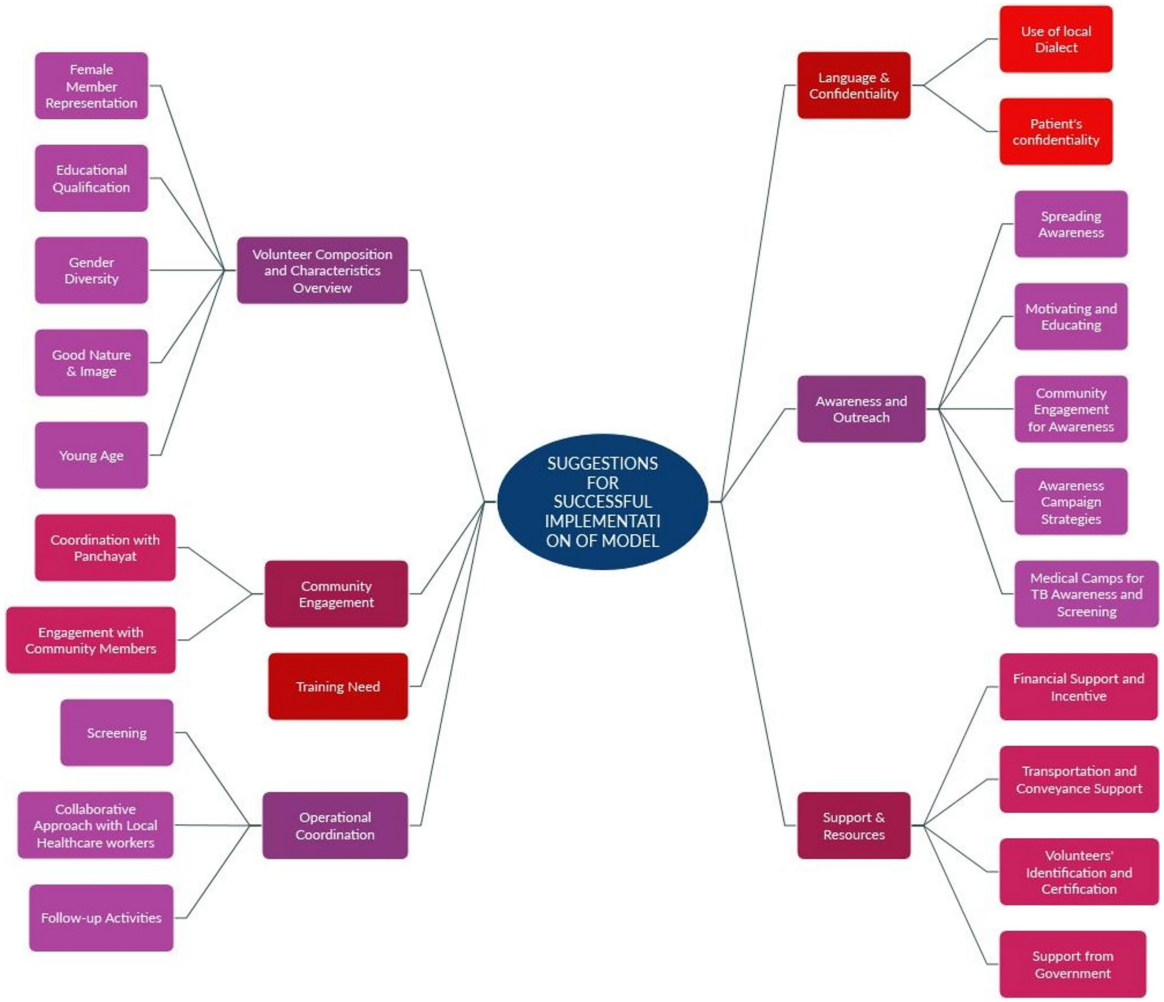
Suggestions for successful implementation of model.

Suggestions span several domains (Figure 6).

#### 4.4.1 Volunteer composition and characteristics overview

This category highlights key volunteer attributes deemed vital for effective engagement in TB-related community health initiatives.

##### 4.4.1.1 Female member representation

Participants from Jaipur and Bhopal emphasized the need for female volunteers, noting their strengths in communication, empathy, and connecting with other women. Their presence fosters inclusivity and enhances outreach, especially in gender-sensitive contexts.

##### 4.4.1.2 Educational qualification

Participants from Ratlam, Pune, Lucknow, Jaipur, and Bhopal advocated for minimum educational standards ranging from 5th to 12th grade, arguing that basic literacy and numeracy enhance volunteers’ ability to comprehend, convey, and record health-related information. Education was seen as improving both competence and credibility.

##### 4.4.1.3 Gender diversity

Beyond female inclusion, participants from Ratlam, Jaipur, and Bhopal stressed the importance of mixed-gender volunteer groups to better address diverse community needs, enhance trust, and navigate cultural sensitivities.

##### 4.4.1.4 Good nature and image

Participants from Ratlam, Pune, Jaipur, and Bhopal highlighted the need for volunteers with a positive demeanour, strong character, and effective interpersonal skills. Health-conscious and socially responsible individuals are seen as role models who inspire community trust.

##### 4.4.1.5 Young age

Youth (18–30 years) were favoured by participants from Ratlam, Pune, Jaipur, and Bhopal for their energy, adaptability, and receptiveness to training. While openness to all age groups was acknowledged, young volunteers were perceived as especially dynamic and effective in implementing programs.

#### 4.4.2 Language & confidentiality

##### 4.4.2.1 Use of local dialect

Participants from Bhopal underscored the importance of using the local language to enhance communication and community understanding of TB-related messages.

##### 4.4.2.2 Patient confidentiality

Participants from Pune and Jaipur emphasized strict confidentiality in handling patient data, viewing it as essential for building trust and ensuring ethical engagement.

#### 4.4.3 Community engagement

##### 4.4.3.1 Coordination with panchayat

Participants from Pune, Jaipur, and Bhopal recommended involving panchayats and community leaders to facilitate communication and resource mobilization.

##### 4.4.3.2 Engagement with community members

Participants from Ratlam, Pune, Jaipur, Lucknow, and Bhopal stressed the importance of community meetings and campaigns to reduce stigma and promote early detection and treatment-seeking behaviour.

#### 4.4.4 Awareness and outreach

Participants from Ratlam, Pune, Patna, and Bhopal offered various strategies to raise awareness.

##### 4.4.4.1 Spreading awareness

Suggestions included awareness drives, media campaigns, and the distribution of educational materials.

##### 4.4.4.2 Motivating and educating

Sessions aimed at demystifying TB and encouraging health-seeking behaviour were recommended.

##### 4.4.4.3 Community engagement for awareness

Community involvement through local leaders and networks was emphasized.

##### 4.4.4.4 Awareness campaign strategies

Participants proposed door-to-door visits, street plays, youth club engagement, and partnerships with NGOs.

##### 4.4.4.5 Medical camps for TB awareness and screening

Camps were widely supported as feasible and impactful for outreach, education, and early diagnosis.

#### 4.4.5 Training needs

Participants from Pune, Patna, Lucknow, Jaipur, and Bhopal highlighted the need for careful selection of candidates for training, culturally sensitive communication strategies, community engagement techniques, TB-specific knowledge, and skills for handling objections. Training must be tailored to the audience’s needs and should enhance volunteers’ communication abilities and confidence.

#### 4.4.6 Support & resources

##### 4.4.6.1 Financial support and incentives

Volunteers should be compensated through stipends or honoraria to ensure sustained participation.

##### 4.4.6.2 Transportation and conveyance support

Especially emphasized by participants from Ratlam, with support from other sites, there was a call for funding travel and logistical support to reach remote areas.

##### 4.4.6.3 Volunteer identification and certification

Participants from Pune, Jaipur, and Bhopal emphasized the importance of ID cards and certification for formal recognition, credibility, and motivation.

##### 4.4.6.4 Support from government

Participants from Ratlam, Pune, Patna, and Bhopal stressed the need for policy backing, infrastructure, and funding to support grassroots initiatives.

#### 4.4.7 Operational coordination

Participants from Ratlam, Pune, Jaipur, and Bhopal identified the following priorities.

##### 4.4.7.1 Screening

Systematic screening of households and high-risk groups was emphasized for early TB detection.

##### 4.4.7.2 Collaborative approach with local healthcare workers

Integration with ASHAs, ANMs, and Anganwadi workers was proposed for referral, outreach, and treatment support.

##### 4.4.7.3 Follow-up activities

Continued support for patients during treatment, including adherence monitoring and home visits, was considered critical for treatment success.

## 5 Discussion

The logic model serves as a visual representation of a program, clarifying the relationships between available resources, activities, outputs, outcomes, and impact. In public health, logic models play a crucial role in planning, implementing, and evaluating programs. Overall, logic models provide a structured framework for program development and evaluation, contributing to more effective interventions and improved health outcomes (11, 13).

Several studies have proposed logic models for tuberculosis (TB) prevention and treatment. These models exemplify a global approach to addressing the complexities of TB control (20–23).

This study developed a logic model for the active screening and motivation of presumptive TB cases by community volunteers registered as members of an organized system under the Ministry of Youth Affairs and Sports, Government of India, who are already involved in various voluntary activities in their respective villages and local areas. This logic model encompasses four key activities, intricately linking available resources (manpower), activities, and outcomes within a structured framework. The model facilitates a comprehensive strategy to manage the complexities inherent in tuberculosis management.

Information, education, and communication (IEC) activities are recognized as a pivotal and primary step in the logic model, as shown in Figure 1, serving as the cornerstone for initiating active TB screening and motivating individuals with symptoms suggestive of tuberculosis. As detailed in the Results section, the meticulously designed NYKS logic model for IEC activities targeting tuberculosis awareness encompasses clear inputs, processes, outputs, and outcomes.

Inputs, such as involving youth members of NYKS in TB-related activities, utilizing IEC materials, providing standard operating procedures (SOP), and employing smartphones for educational videos, ensure a comprehensive approach to addressing the multifaceted challenge of tuberculosis. The subsequent processes involve regular activities for the general population and more frequent sessions for vulnerable groups, as shown in Figure 2, catering to diverse audience needs in their respective areas and villages.

The outputs, measured by attendance and participation, gauge the immediate impact of these activities. Meanwhile, outcomes, measured by the number of individuals made aware of tuberculosis, reflect the ultimate goal of these initiatives. This systematic approach not only facilitates efficient resource allocation but also supports continuous improvement in tuberculosis awareness efforts.

Numerous studies underscore the critical role of IEC activities in the effective management of tuberculosis. The positive influence of IEC activities has been reported to extend beyond individuals to the community level, fostering enhanced engagement in TB control efforts in India and abroad (24–26). For instance, a study in Odisha, India, revealed that sensitization activities not only improved Interpersonal Communication Skills (IPC) but also bolstered community confidence in managing TB (25). The Central TB Division’s guidelines for programmatic management of TB preventive treatment in India also include IEC materials as part of the National TB Elimination Program activities, emphasizing the strategic importance of information, education, and communication initiatives in the broader context of tuberculosis control (27).

The second activity in the NYKS logic model involves NYKS youths undertaking screening activities, positioning them as pivotal measures following IEC efforts to systematically identify and assess individuals as potential TB cases (Figures 1, 2). The logic model for screening activities targeting presumptive tuberculosis (TB) patients is meticulously crafted with a focus on key inputs, processes, outputs, and outcomes to enhance the effectiveness of TB management. Furthermore, the incorporation of key indicators across domains ensures systematic and comprehensive oversight, facilitating structured evaluation of the screening activities.

Numerous studies underscore the significant benefits of screening activities in the effective management of tuberculosis (TB). The WHO recommends systematic screening for TB because it not only benefits individuals but also extends its impact to the broader community by reducing TB prevalence and preventing future disease (28). The Centre for Disease Control and Prevention (CDC) recommends targeted screening, particularly for high-risk groups, such as those in close contact with TB patients, immunosuppressed individuals, and healthcare workers (29). A systematic review affirmed that TB screening plays a crucial role in identifying patients earlier in their clinical course, consequently enhancing their clinical outcomes (26). In summation, these studies collectively emphasize the pivotal role of screening activities in TB management, showcasing their potential to reduce TB prevalence, identify cases earlier, improve health outcomes, and mitigate tuberculosis transmission within the community.

The subsequent activity in the logic model focuses on case referral, underscoring its importance as a sequential process following screening, aimed at directing identified potential TB cases towards diagnostic, therapeutic services, and management. The referral activity for presumptive tuberculosis (TB) cases is characterized by a systematic and well-structured approach. Through comprehensive training, these NYKS community volunteers are equipped with the necessary knowledge of the case referral process, including the use of referral slips and data registers, enhancing their effectiveness in facilitating referrals. The process domain entrusts these volunteers with the crucial responsibility of referring presumptive TB cases for medical check-ups, emphasizing the role of grassroots engagement in the healthcare system. The quantifiable output of this activity, measured by the number of presumptive cases referred with accompanying slips, provides tangible metrics for the success and reach of the referral process. The outcome domain assesses the impact by evaluating the number of referred cases that ultimately consult for medical examinations, shedding light on the efficacy of the referral system in facilitating timely healthcare access.

The final activity in the logic model centres on case detection, emphasizing its critical role as the concluding stage following case referral activities, ultimately leading to the identification and confirmation of tuberculosis cases for prompt intervention and treatment. In the case detection phase of the tuberculosis (TB) intervention, a crucial emphasis is placed on empowering volunteers with smartphone access for diagnostic data collection. The “input” domain ensures that these volunteers have direct contact details for the Medical Officer (MO) and Laboratory Technician (LT), establishing a foundation for streamlined communication and collaboration. The subsequent “process” domain underscores the primary responsibility of volunteers in obtaining medical check-up/investigation results and collecting updates on enrolled cases from the Directly Observed Treatment, Short-course (DOTS) provider regularly. The measurable “output” of this activity is determined by the total number of cases identified as positive for TB, providing a tangible indicator of the intervention’s impact at the ground level. The “outcome” domain evaluates the broader impact of the case detection activity by comparing the TB case detection status against the baseline rate, providing insights into the effectiveness of the intervention over time.

A typical Indian village represents a conglomeration of different micro-climate zones, local terrains, and socio-economic communities. Youth club members come from diverse backgrounds, representing every part and corner of the village. The public health system of India employs accredited social health activists (ASHA) as the most peripheral health workers at the village level. Auxiliary nurse midwives (ANM), another type of peripheral health worker in India, provide services to about 2–5 villages through health sub-centres. Given the diverse heterogeneity in any typical Indian village and the limited availability of peripheral health workers, it is prudent to involve community members representing various characteristics of the village in TB screening. NYKS youth club members emerge as such community members, coming from diverse backgrounds and different parts of the village. Because of their diversity and proximity to the community, NYKS youth club members will have greater acceptability among community members and better penetration for outreach activities. These are the reasons why NYKS youth club members have an advantage over others, as a key input in the developed NYKS logic model.

Qualitative findings revealed a range of attitudes towards the proposed model. Most participants expressed strong acceptance and enthusiasm for it, highlighting its feasibility and relevance across diverse community and institutional settings.

Participants emphasized the importance of involving local NYKS volunteers, acknowledging their potential to engage communities effectively. The recognition of youth as influential motivators further underscored the role of community empowerment in TB control. Willingness to collaborate signalled openness to community-based interventions.

Nonetheless, acceptance was not universal. A minority expressed conditional acceptance, contingent on specific contextual or logistical factors. Others voiced tentative disagreements or doubts, often due to uncertainty about the model’s effectiveness or concerns about duplication with existing health structures. A few participants strongly disagreed, primarily questioning the need for a separate survey team and expressing trust in the roles of current dedicated health workers. Some also reported uncertainty, indicating a need for clearer information and improved stakeholder communication.

Overall, the findings suggest a broadly favourable view of the model, tempered by nuanced concerns. Similar acceptance of community-driven TB interventions has been documented in other settings (30–32).

These findings reaffirm the value of leveraging community-based structures for TB control efforts. However, addressing stakeholder concerns and ensuring sustained communication will be critical for successful implementation in the Indian context.

Several key reasons underpinned the widespread acceptance of the model, reflecting a convergence of operational practicality and community values. These included the presence of a local implementation unit, alignment with health priorities, reinforcement of the healthcare system, and the model’s perception as a form of social service.

Participants frequently cited trust in local volunteers as a primary factor. Their familiarity with community dynamics enabled personalized counselling and easier access to services. The significance of local implementation units has also been highlighted in other studies (33, 34).

Another reason was the model’s alignment with TB as a critical health priority. Participants viewed TB as a serious local concern and saw the intervention as an opportunity to address it effectively. By improving awareness, prevention, and access to treatment, the model was regarded as a community asset. Similar themes emerged in studies from Bihar and elsewhere (33, 35).

Strengthening the healthcare system was another motivator. Participants anticipated that additional volunteer support would enhance efficiency in active case finding, referrals, and access to diagnostics. House-to-house visits and the proximity of testing services were seen as valuable additions. Similar findings were observed in Bihar and Haryana (35, 36).

Finally, many participants perceived the initiative as a form of social service. This view was rooted in a broader ethos of social responsibility and collective welfare. The model was embraced as a means of giving back to the community. Similar findings have been reported from Tamil Nadu and Bihar, where community participation was driven by a sense of civic duty and compassion (31, 35).

In summary, the model’s acceptance was shaped by its integration within the community, alignment with health system priorities, and perceived social value. Emphasizing these elements can enhance future community-based TB programs in India.

Despite the general acceptance, participants identified several barriers that may impede the model’s implementation. These included financial, logistical, community trust, and societal discrimination-related barriers.

Financial barriers emerged as significant constraints. Many stakeholders were unaware of government incentives for TB volunteers. Concerns about competition with ASHA workers for incentives also emerged as a complicating factor. For economically vulnerable patients, the cost of care—even when subsidized—remained burdensome. Most notably, the lack of financial support for NYKS volunteers undermined their motivation and sustained engagement. These challenges echo findings from South Africa, Nigeria, and Indonesia, where financial disincentives led to high volunteer attrition and weak program continuity (37–39). The Myanmar study also highlighted the importance of financial support in community-based TB care (32). A unique contribution of this study was the insight into intra-system competition for incentives, revealing complex dynamics between different cadres of health workers.

Logistical challenges were also reported. *Difficulties in transporting older adult patients, limited presence of NYKS workers in some villages, and infrequent interaction with communities weakened the program’s visibility and continuity*. Concerns were also raised about NYKS workers’ lack of medical training and their perceived redundancy compared to established health staff. These concerns mirror findings by Vyas et al. from tribal regions, where logistical hurdles reduced access to care (34). Similar operational difficulties were documented in the Nigerian and Indonesian contexts (38, 39). The scepticism regarding role overlap suggests a need for clarifying task boundaries and improving integration with existing systems.

Community trust barriers centre on issues of resistance, denial, fear of stigma, and reluctance to share health information. Such attitudes limit the effectiveness of outreach and screening activities. The Indonesian study similarly emphasized the importance of trust-building strategies for community engagement (39).

Societal discrimination also emerged as a significant barrier. Reports of caste-based exclusion and social status-related prejudice hindered access to healthcare and undermined NYKS volunteers’ efforts, particularly among marginalized groups. These challenges align with prior Indian studies that identified stigma and discrimination as key barriers to individualized follow-up and health system responsiveness (33). Tackling these issues will require culturally sensitive, equity-oriented approaches to ensure inclusive health service delivery.

In conclusion, addressing these barriers is essential for operationalizing the NYKS model. This includes increasing awareness about incentives, fostering coordination among health actors, strengthening logistics, enhancing community trust, and combating structural discrimination. By comprehensively addressing these concerns, the model can achieve its intended impact on TB control and community health.

The suggestions offered by participants provide valuable insights into enhancing the NYKS model for tuberculosis (TB) awareness and prevention. These span several key domains, including volunteer composition, community engagement, language use, training needs, support, and operational coordination.

The inclusion of female volunteers was seen as critical for community outreach, especially in gender-sensitive contexts. Educational qualifications enhanced volunteers’ competence and credibility in conveying health information. Gender diversity and youth volunteers contributed to dynamic and inclusive engagement. Studies validate the need for diverse, well-trained, and gender-diverse volunteers to improve TB control programs (31, 34, 36, 40). This study highlights the added strength that female volunteers bring to community engagement and health outcomes.

The use of the local dialect and assurance of confidentiality were deemed essential. Local language improved the understanding of TB messages, while confidentiality built trust—crucial for successful treatment engagement. Previous studies support these findings, emphasizing effective communication and the role of trust in TB control (34, 38).

Effective community engagement relied on coordination with local governance structures and active participation from community members. Involving panchayats and local leaders enhanced communication and resource mobilization. Community meetings, awareness campaigns, and interactive sessions fostered proactive TB prevention efforts. Several studies confirm the importance of governance involvement in TB control programs (31, 32, 34, 36).

A multifaceted approach—incorporating media campaigns, health motivation, and screening camps—was considered vital. Cultural events and collaborations with NGOs further strengthened outreach efforts and improved TB detection. Previous research also underscores the value of community-based interventions and multifaceted strategies (32, 34).

Tailored training is essential to equip volunteers with the skills needed for effective community engagement. Training programs should be context-specific and emphasize sensitive communication strategies. Studies confirm the role of tailored training in improving volunteer performance (39, 40).

Financial incentives, transportation support, volunteer certification, and government backing were considered vital for sustaining volunteer engagement. Adequate incentives and logistical support were viewed as essential for program sustainability. Studies corroborate the need for robust support systems to maintain volunteer participation and success in TB control (32, 34, 37).

Operational coordination requires systematic screening, collaboration with healthcare workers, and consistent follow-up activities. Targeted screening and regular follow-ups improved TB detection and treatment outcomes. Studies confirm the effectiveness of volunteer involvement in TB case finding (31, 34, 36).

Incorporating these suggestions into the NYKS model can enhance its acceptability and effectiveness in addressing TB awareness and prevention. However, successful implementation will require coordinated planning, adequate resources, and sustained stakeholder engagement.

### 5.1 Strengths and limitations of study

The study brings together the public health system, Nehru Yuva Kendra Sangathan, and the community for a common cause: tuberculosis. This is a major strength of the study. Logic model development followed a standard procedure, which also adds strength to it. However, there are a few limitations as well. The actual feasibility of implementing the logic model needs to be assessed in program settings through empirical testing or validation of the proposed model. The effectiveness of this model compared with other conventional models also needs to be evaluated in program settings.

This study has several strengths that enhance its methodological rigor. First, adherence to the Consolidated Criteria for Reporting Qualitative Research (COREQ) ensured transparent and systematic reporting. Second, the multi-staged sampling strategy and use of data saturation principles improved the representativeness and depth of the findings. Third, structured tools—such as interview guides and questionnaires—ensured consistency and alignment with the study objectives.

However, the study also has limitations. Participant selection may introduce bias, affecting the generalizability of the results. The interpretive nature of qualitative analysis poses a risk of researcher bias, underscoring the need for reflexivity. Additionally, social desirability bias may have influenced participants’ responses, especially regarding community engagement and the acceptability of the intervention model, despite efforts to ensure cultural sensitivity.

## 6 Conclusion

This study develops a logic model for the active screening and motivation of individuals with TB suggestive symptoms and other vulnerable populations in their local community with the help of members of Nehru Yuva Kendra Sangathan (NYKS). Involving the existing group of youth members who are registered under NYKS as volunteers in the TB screening program for early TB case detection would assist the National TB Elimination Programme in strengthening its case-finding activities. The effort will also empower the community to seek early medical intervention by gaining knowledge about TB through regular interaction with local youth. This would also set an example of resource convergence for empowering the community and tackling one of the most severe health problems in the country.

As this study assessed feasibility and acceptability only, the findings should be interpreted as preliminary and not as evidence of the model’s direct impact on TB outcomes. The findings of this study highlight the acceptance of the NYKS model for active TB screening and motivation among diverse stakeholder groups in India. While there is widespread acceptance of the model, several barriers need to be addressed to ensure successful implementation. Financial challenges, logistical issues, concerns about community engagement, and societal discrimination emerge as key barriers that require targeted strategies for mitigation.

The study underscores the importance of community engagement, alignment with health priorities, and the perception of the model as a form of social service in promoting acceptance and feasibility. Recommendations for future research include exploring gender dynamics, evaluating cultural strategies, conducting longitudinal studies on program sustainability, and investigating strategies for mitigating discrimination.

By incorporating these recommendations into future research and practice, community-based TB control programs can enhance their effectiveness, reach, and sustainability, potentially contributing to the broader goal of eliminating TB in India and promoting community health and well-being.

## Data Availability

The original contributions presented in the study are included in the article/supplementary material, further inquiries can be directed to the corresponding author.
